# The Effects of Pain, Agitation, Delirium, Immobility, and Sleep Disruption Education on Novice Nurses in Adult Intensive Care Units

**DOI:** 10.3390/healthcare10081538

**Published:** 2022-08-14

**Authors:** Szu-Ying Lee, Chieh-Yu Liu, Te-Yu Wu

**Affiliations:** 1 Department of Nursing, Mackay Medical College, New Taipei City 25245, Taiwan; 2 Department of Health Care Management, National Taipei University of Nursing and Health Sciences, Taipei City 112303, Taiwan

**Keywords:** adult intensive care unit, clinical ladder, PADIS, education intervention, novice nurse

## Abstract

Intensive care unit (ICU) patients experience highly complex health problems, such as pain, agitation, delirium, immobility, and sleep disruption (PADIS), and require professional nursing care. The assessment of PADIS is critically important for ICU nurses, and therefore, PADIS education programs need to be conducted for these nurses to update and improve their caring knowledge, attitudes, and skills. The aims of this study are to bridge this gap by evaluating the effects of PADIS education programs on the knowledge, attitudes, and skills of these nurses, and compare the difference between novice and advanced nurses after receiving the PADIS education programs over a short period of time. In this quasi-experimental study, 112 nurses in ICUs were recruited by researchers and participated in the PADIS education programs. The PADIS education intervention was performed in a teaching hospital in Taipei. A demographic and self-developed PADIS care knowledge questionnaire was used. A baseline (T1) was measured before the interventions, followed by post-test (T2) immediately after the programs, and subsequently a follow-up (T3) test one month later. The results indicated that knowledge and skill scores between novice and advanced nurses varied significantly in T1 but not in T2 and T3. Thus, education programs can significantly assist novice ICU nurses to improve their short-term knowledge, attitudes, and skills, and PADIS education programs are strongly suggested for clinical nursing practice.

## 1. Introduction

Patients in intensive care units (ICUs) have highly complex health problems, such as pain, agitation, delirium, immobility, and sleep disruption (PADIS), and need professional nursing care. Pain, agitation, delirium, immobility, and sleep disruption are common health problems in the ICU, which result in negative patient outcomes [1]. Pain results in patients’ sleep deprivation and exacerbates delirium and agitation [2]. Delirium is also an important factor impacting the morbidity and mortality of patients [3]. Poorer sleep quality and sleep disruption are also important issues for patients due to environmental stimuli or physical discomfort [4]. These health problems need professional nursing care in order to improve health conditions and increased the quality of patients’ care. However, a previous study found that nurses had insufficient knowledge regarding pain management; the average score for pain knowledge was 63.28 points (out of 100 points), and the average correct answer rate was 63.1% [5]. In addition, in Rowley-Conwy’s study, nurses were found to lack knowledge on delirium assessment and management [3], and they lack the knowledge and right attitude regarding the use of physical restraints and do not understand the impact of immobility [6]. The level of nurse professional capability also impacts the quality of care and health outcomes. Therefore, the clinical nursing ladder system is a strategy for improving nurses’ professional abilities.

The clinical nursing ladder system was established over several decades [7], with the purpose of advancing nursing professional competency, improving job satisfaction, and also providing quality care for patients [8,9,10]. There are four levels of the clinical ladder in the nursing system: Level 1 is defined as newly employed nurses (N1), nurses in level 2 (N2) need to accomplish clinical professional ability training and examinations, and level 3 (N3) indicates proficient nursing capabilities that include decision making and playing an educator role; nurses in this level must pass various training interventions and examinations in the long term. Level 3 nurses are identified as advanced nurses, who can then move to level 4 (N4) as expert nurses [11]. The development of advanced nurses requires a long-term training program and cultivation. However, there are 86% novice nurses (N1 and N2) and 14% advanced nurses (N3 and N4) in the nursing field, based on a national survey in Taiwan [11]. Thus, novice nurses occupy most of the total population.

A previous study revealed that 80% of nurses believed it was difficult to pass the requirements of N3 [12]. Therefore, there is a large difference in the proportion of novice nurses relative to advanced nurses. According to a previous study, advanced nurses were more likely to have a positive belief in knowledge, attitudes, and skills in implementing evidence-based practice than novice nurses [11]. Advanced nurses tend to be older, have more working experience, and have a higher educational level [11]. Therefore, enhancing critical care ability appears to be important for novice nurses.

Novice nurses must face both the rapid changes in patients’ disease conditions and the complexity of caring for patients in a critical care setting. However, many novice nurses lack confidence in their critical care nursing skills and also have insufficient knowledge to work in an ICU [13,14]. In addition, novice nurses were not well-prepared to transition to the professional role in an ICU setting [14]. Unfamiliar knowledge and skills might result in a risk to ICU patients’ safety. Therefore, clinical nurses’ professional competency might be enhanced through various educational programs. According to a study by Santana-Padilla et al. [15], educational programs can improve clinical nurses’ knowledge and skills and also change the nurses’ care behavior, thus improving patient outcomes.

In order to provide comprehensive nursing assessments and professional care, PADIS clinical guidelines were appropriate for ICU patients’ care. The Society of Critical Care Medicine (SCCM) proposed the updated pain, agitation, and delirium (PAD) clinical guidelines in 2013 [16]. However, immobility and sleep disruption are also important health problems for ICU patients and, therefore, the guidelines of these two sections were updated and expanded as PADIS in 2018 [17]. The PADIS clinical guidelines are comprehensive and structured assessment guidelines for assessing ICU patients’ health problems in the ICU [17]. The Taiwan Society of Critical Care Medicine developed the Taiwan version of the PADIS clinical guidelines, with reference to past studies, to improve the nurses’ ICU care ability and update their knowledge [16,17]. However, the clinical knowledge of PADIS does not appear to be adequately implemented in the ICU. A previous study indicated that ICU nurses still receive outdated treatment knowledge regarding caring for patients, and even though the pain, agitation, and delirium (PAD) international guidelines have been implemented, nurses also have insufficient or inconsistent knowledge and understanding of applying PAD [18,19,20].

According to the comprehensive literature review above, there is still a knowledge gap regarding PADIS guidelines in ICU clinical practice.

Few studies have explored the effectiveness of PADIS education programs on the knowledge, attitudes, and skills of novice ICU nurses. Studies on comparing the differences between novice and advanced nurses regarding these three domains in PADIS education programs are still limited. Therefore, this study aimed to bridge this gap by providing evidence of the effects of PADIS education programs on the knowledge, attitudes, and skills between novice and advanced nurses after receiving PADIS education programs. The implementation of PADIS education programs might enhance the quality of ICU patients’ care, especially for novice nurses. As a result, they may develop a similar level of professional knowledge, attitudes, and skills to advanced nurses before accomplishing the clinical nursing ladder requirements.

## 2. Materials and Methods

### 2.1. Participants and Study Design

In this study, a quasi-experimental research design was used, that included pre- and post-testing and purposive sampling. Participants underwent a joint PADIS education program. Data were collected at three time points as follows: at pre-test (T1), immediate testing after the education program (T2), and a follow-up test one month later (T3). The researchers proposed a PADIS education curriculum plan to five adult ICU supervisors in April 2019. The PADIS education curriculum was announced on bulletin boards in five ICUs, and ICU nurses were also informed by ICU supervisors two weeks before the curriculum. ICU nurses were invited and encouraged to take part in the PADIS education curriculum. All participants were volunteers and provided informed consent. 

Participant inclusion criteria were nursing staff (1) in intensive care units who had worked there for three months and above, (2) had a registered nurse license or nurse license, and (3) were willing to take part in the study. Participants who had only worked in pediatric intensive care units or emergency rooms were excluded from the study. The researchers explained the study purpose and individual rights and obtained written consent from all the participants. 

The participants were classified into two categories, based on the clinical ladder system. In this system, nurses in level N2 and below were identified as novice nurses, while those in level N3 and above were in the advanced nurse group. The levels of nursing proficiency were defined by Benner in 1982; the process of advancing from a novice nurse to an expert one depends on professional ability, working experience, administrative abilities, and educator capability [21]. An expert nurse has more than five years of working experience based on Benner’s definition. In Taiwan, the levels of nursing proficiency are based on Benner’s theory. In our study, the level of nursing proficiency was defined by Taiwan Nurses Association (TWNA), and the advanced system is named the nursing ladder system [22]. N, N1, and N2 are defined as less than 1 year to 2 years of working experience; in terms of academic competence, completing a qualified reading report is required in N1, whereas a qualified case study report or evidence-based nursing report is required in N2. N3 and N4 levels are defined as having 3 years and above of working experience and also completing a qualified project report or research paper in terms of academic competence [22]. Each level of qualified nurse has to adopt and pass examinations and training programs. According to the requirements of TWNA, N to N2 levels are classified as novice nurses, and N3 and N4 are classified as advanced nurses based on working experience and academic ability [11,22]. However, nurses with more working experience were not equal to those passing the certification of the Taiwan Nurses Association (TWNA) because they did not accomplish the requirements of teaching and academic abilities.

### 2.2. Measurements

A demographic questionnaire and a self-developed PADIS care knowledge questionnaire were used in this study and included three domains: knowledge, attitudes, and skills. The PADIS care knowledge questionnaire comprised 15 items, and the total score ranged from 15 to 75 (each question was answered on a 5-point rating scale). A higher score indicated better PADIS knowledge and skills. 

The domain of knowledge included seven items: “I have heard the PADIS assessment guidelines before”, “I know the PADIS assessment guidelines very well”, “I am very familiar with the implications of the PADIS assessment”, “I realize the methods of PADIS assessment”, “I understand the importance of this course for patients’ care”, “I know the principles of applying of PADIS guidelines”, and “I have the correct PADIS knowledge to assess patients” The domain of attitude contained three items: “PADIS education is very helpful for my nursing work”, “I would like to share the content of PADIS education to other medical staff”, and “I think PADIS assessment is benefit for patients’ health outcomes”. Regarding the domain of skills, it included five items: “I can apply the PADIS assessment guidelines in my daily patient care process”, “My nursing care skills can be improved by PADIS education”, “I can revise my past care strategies based on the PADIS assessment outcomes”, “The quality of patient care can be advanced by PADIS education”, and “I can apply the PADIS guidelines for each assessment item correctly”.

Regarding the validity and reliability of the questionnaire, the self-developed PADIS care knowledge questionnaire was examined for content validity by five experts with more than 10 years of ICU clinical experience. The content validity was 0.9, and the Cronbach α was measured at 0.89 for internal consistency. Self-administered questionnaires for the pre- and post-intervention assessments were completed, and a follow-up questionnaire was conducted one month later to test the effectiveness of the interventions over a period of time. The interventions were conducted between May and September 2019.

### 2.3. The PADIS Education Intervention

The Taiwanese version of the PADIS clinical guidelines was modified in 2018 according to the American Society of Critical Care guidelines, and it was formatted by Taiwanese ICU experts into an easily understandable version. A total of five necessary sections were conducted, and the content of the education program included the definition of PADIS, assessment tool, medication, and non-pharmacological strategies. There were 40 to 50 min lectures in each section, an hour of group discussion, and assessment tool practices. Two researchers and one ICU head nurse were involved in planning the PADIS education program, and one senior ICU nurse was a nursing instructor and delivered the intervention. The entire intervention education program was delivered by one senior nursing instructor in order to ensure consistency in the content of the intervention process. The teaching materials included PowerPoint, video, and the PADIS clinical care guide booklet. In addition, clinical guideline reminder cards were placed around patients’ bedsides to remind ICU nurses about the practical implementation of these guidelines.

### 2.4. Statistical Analysis

IBM SPSS Statistics for Windows (Version 24.0) was used to analyze the data. Continuous variables are expressed as means (M) and standard deviations (SDs), and categorical variables are expressed using frequency (*n*) and percentage (%). Independent *t*-tests and chi-square tests were performed to determine whether there were significant differences in the PADIS questionnaire regarding demographic variables. Repeated-measure ANOVA was applied for investigating the difference between novice and advanced nurses. The two-tailed significance level was *p* < 0.05.

### 2.5. Ethical Considerations

The study was approved by the institutional review board of MacKay Memorial Hospital (IRB No:19MMHIS242e). The research was conducted with data collection starting after receiving written consent from the participants. This research was supported by the Taiwan Nurses Association (TWNA-1091011).

## 3. Results

### 3.1. Description of Demographic Characteristics

A total of 112 ICU nurses were recruited, and there was no attrition rate in the follow-up period. The participants’ average age was 29.9 years (SD = 8.4 years), 90.2% had a university degree and above, 58% had over three years of working experience in the ICU, and 46.6% had under five years of working experience in the nursing field. Regarding the clinical ladder level, the majority of participants were on level N1 (45.5%), and 15.2% were on level N3 or N4. The data regarding the demographic characteristics of all participants are shown in Table 1.

As shown in Table 2, the participants were classified into two categories, based on the clinical ladder system. The mean age had varying values and standard deviations in the two groups (27.66 ± 2.56 vs. 42.47 ± 3.41). The majority of those in the novice nurse group had a university degree (86%), and 51% had an ICU working experience of over three years. In the novice nurse group, a total of 37% had over five years of nursing working experience, and 68% had an ICU license. In the advanced nurse group, 76% had a university degree, and all had over three years and over five years of ICU working experience.

Our evaluation of the associations between the clinical ladder and sociodemographic features indicated significant relationships between age, gender, marital status, education background, ICU working experience, and nursing working experience.

### 3.2. The Effects of the PADIS Education Program

Table 3 presents the differences in PADIS scores between advanced nurses and novice nurses at T1, T2, and T3. The mean score for PADIS questionnaires increased across the three time points during the study period. At T1, the advanced nurses’ scores were significantly higher than those of the novice nurses (Mean ± SD = 52.76 ± 10.33, 40.33 ± 16.44, *p*-value = 0.006). Regarding the knowledge, attitudes, and skills subscales, the advanced nurses’ scores were also higher than those of the novice nurses (Mean ± SD = 23.11 ± 5.27, 17.23 ± 7.95 for knowledge; Mean ± SD = 11.41 ± 2.73, 9.48 ± 3.75 for attitudes; Mean ± SD = 18.23 ± 3.57, 13.62 ± 5.98 for skills). Further, the results showed that the knowledge and skill scores were significantly different between novice and advanced nurses at T1 (*p* = 0.013 in knowledge and *p* = 0.002 in skills). However, there were no significant differences in attitude between the two groups at T1. 

The differences between novice and advanced nurses were further investigated using repeated-measure ANOVA in a general linear model (GLM) after adjusting for age and marital status. The results are shown in Table 4. From Table 4, it can be derived that the PADIS total score, knowledge score, attitude score, and skills scores showed similar patterns: the scores showed statistically significant differences at T1 (pre-test); however, the results showed non-significant differences at T2 (first post-test) and T3 (second post-test).

**Figure 1:**
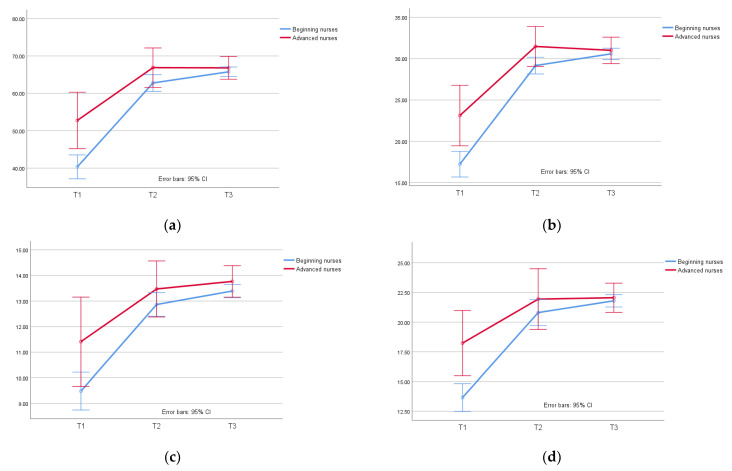
The results of PADIS scores for novice and advanced nurses: (**a**) PADIS scores; (**b**) knowledge scores; (**c**) attitude scores; (**d**) skills scores.

Figure 1 illustrates the trends of the follow-up PADIS scores for the two groups. The mean score of PADIS and the subscales are listed in Table 2, according to which the overall trends increased during the three points in time. A comparison of the two groups revealed that the advanced nurses’ group scores were higher than those of the novice nurses in T1 and T2. However, the two groups presented a very similar trend in the T3 follow-up, as well as in knowledge, attitudes, and skills.

## 4. Discussion

### 4.1. Demographic Characteristics

The present study is the first to explore the possible effectiveness of PADIS educational interventions in enhancing knowledge, attitudes, and skills among novice nurses in adult ICUs. The majority of participants were female, with an average age of 29.8 years. The findings were similar to those reported in a study by Min and Kim [23] in Korea. However, in that study, 50.3% of the ICU nurses were aged 26–30 years in Korea [23], while in our study, the participants were younger (66.1% were aged 20–29 years). This could be due to the different geographical study regions. Consistently, more than half of the nursing staff in acute or critical care settings were younger. In Ajri-Khameslou’s study [24], the mean age of ICU nurses was 33.76 years, and 52.1% of ICU nurses were aged from 31 to 40 years old; thus, the age group was older than that in our study. This difference might be attributed to the nurses’ working environments and cultural diversity.

In addition, 90.2% of the participants had a university degree. These findings were similar to those of Weng [8]. The possible reason might be that the nursing university in Taiwan is universal. Regarding nursing working experience, 46.4% of the nurses had over five years of nursing working experience in our study, compared with Min and Kim’s study [23], where 8.4% of nurses had less than five years of working experience, whereas 28.8% had five to ten years of experience in acute or critical care settings. The difference in results might be because the population in our study was only recruited from adult ICUs as opposed to emergency rooms, operation rooms, or pediatric ICUs. 

An issue of concern in this study was that only 16% of the nurses were advanced nurses; however, 90.2% had a university degree. This could be because clinical nurses may have less willingness to participate in the nursing clinical ladder program. According to Li et al. [25], most clinical nurses showed less willingness to participate in the nursing clinical ladder program, because the case-report writing requirement may be more difficult for them than caring for patients and could result in additional mental stress or impact their quality of life [25]. Being an advanced nurse requires spending a considerable amount of time enhancing novice nurses’ abilities to enable them to become expert nurses [6]. Therefore, nurses with more working experience do not necessarily hold a higher position on the clinical ladder. Clinical nurses should be encouraged to participate in the clinical ladder program and receive counseling resources or strategies to improve their job satisfaction and achievement. 

### 4.2. The Effects of the PADIS Education Program

In our study, the results showed that there were significant differences in the knowledge and skill scores between novice and advanced nurses in T1; however, knowledge, attitudes, and skills were not significantly different in T2 and T3. This could be because there was consistency between the two groups of ICU nurses regarding their positive attitudes toward providing patient care. This finding aligns with that of Wang et al. [26], where most of the ICU nurses had positive attitudes towards patient outcomes, such as getting out of bed early to avoid the complication of immobility [26]. The ICU nurses significantly improved their PAD assessment ability regarding ICU patient care after the PAD education intervention [27]. 

There was a significant difference between the two groups during pre- and post-test after the educational interventions. This result was similar to that reported by Eskandari et al. [28]. The studies of Ramoo et al. [29] and Rowley-Conwy [3] also revealed that critical care nurses had improved knowledge of delirium assessment after educational interventions, consistent with the current study. The findings of Eskandari et al. also supported that knowledge, attitude, and intention significantly influenced nurses’ practice in using physical restraints [28]. A PADIS education program was effective for novice nurses to improve their knowledge and skills in the ICU in the short term. This finding was similar to that of Yeh et al. [30]. In addition, there were no significant differences in the post-intervention and one-month follow-up in this study. The learning effectiveness of the intervention was still retained one month later for the novice nurses’ group. This finding was similar to that of Chyan et al. [31]. Continuing education can, thus, improve the knowledge, perception, and attitudes of novice nurses regarding reducing patients’ restraints in the ICU [30]. Therefore, this study revealed the importance of continuing education for ICU nurses, especially novice nurses, to improve their clinical abilities. 

Pharmacological and non-pharmacological treatments are necessary for patients’ health outcomes. Delirium is associated with critically ill adults and results in the inability of patients to think clearly in the short term. Patients may experience a delirium shift between clear thinking and agitation and/or drowsy and confusion. Lack of sleep, pain, physical restraint, a noisy environment, and the use of sedatives and strong analgesics are contributing factors [32]. Some pharmacology nursing care is necessary for patients experiencing delirium. The use of continuous intravenous infusion of midazolam, propofol, and morphine to induce sedation in mechanically ventilated patients in the ICU resulted in a lower rate of delirium than the use of medications. Furthermore, nurses should assess patients for brain function assessment twice daily using CAM-ICU and RASS [32,33]. Thiamine is necessary for energy metabolism, and critically ill patients are particularly at risk of developing thiamine deficiency and related complications to delirium [34]. Considering the low side effects and cost, this treatment strategy might provide a better alternative to other medications used to prevent and treat delirium. Currently, there are no standards regarding prescription dosage, the timing of supplementation, and frequency of administration for thiamine [34]. 

In addition to the use of medications, non-pharmacological nursing strategies should be considered, such as environmental noise reduction, eye masks, music, ear plugs, and encouraging early mobility, which might reduce the incidence of delirium through circadian rhythm [33,35]. Family members need to be involved in the therapeutic process, as it is helpful for family members to increase their perception of the work performed by the medical staff and provide mental support for patients. Additionally, cognitive exercise may significantly decrease the incidence and duration of delirium in critically ill patients; it can improve their mobility, self-confidence, psychological status, and belief in recovery, as well as shorten their length of hospital stay, facilitate their return to society as soon as possible, and improve their quality of life [36].

This study has several limitations. First, there was inequality in the sample size for the two groups of novice and advanced nurses. Although there was a significant difference in the number of participants, there were actually few advanced nurses in the clinical nursing population used in this study. In addition, all the participants were recruited from a hospital in Taipei City; therefore, the findings might not be representative of other districts. Second, in terms of the limitation of research measurement, the 15-item self-administrated of PADIS care knowledge questionnaire needs to be developed with more comprehensive items for evaluating knowledge, skills, and attitudes in future research. Exploratory factor analysis (EFA) and confirmatory factor analysis (CFA) need to be used for verifying the PADIS care knowledge questionnaire in further studies. Third, the PADIS program was conducted to test its effects on improving the knowledge and skills of novice nurses in the short term; however, some potential confounders, such as reviewing lecture contents individually or taking part in additional private relevant training might not have been included in this analysis. Fourth, a long-term follow-up study needs to be conducted in order to explore the learning delay effect in the future. Nevertheless, the results of this study may have implications for improving ICU care capability for novice nurses in the short term.

## 5. Conclusions

PADIS nursing education programs can significantly assist novice adult ICU nurses to improve their knowledge, attitudes, and skills in the short term. In addition, the interventions may also narrow the knowledge gap between novice and advanced nurses and improve adult ICU care competency over a short period of time. Therefore, the PADIS clinical guideline educational programs should be applied in other acute care settings, and ICU nurses should be encouraged to adapt the knowledge during practical patient care. In addition, patients’ outcomes should be evaluated after conducting training on the clinical guidelines to provide high-quality care for patients. Therefore, the PADIS education program is strongly suggested for clinical nursing practice.

## Figures and Tables

**Table 1:** Characteristics of the study participants (*n* = 112).

Variables	*n*	%
Age (M ± SD)	29.9 ± 8.4	
Gender		
Male	7	6.3
Female	105	93.8
Age		
20–29	74	66.1
30–39	20	17.9
40–49	13	11.6
50–59	5	4.5
Marital status		
Single	95	84.8
Married	17	5.2
Education Background		
College	11	9.8
University	95	84.8
Master	6	5.4
ICU working experience		
0–1year	19	17
1–2 years	17	15.2
2–3 years	11	9.8
3 above	65	58
Clinical Ladder		
N	15	13.4
N1	51	45.5
N2	29	25.9
N3	16	14.3
N4	1	0.9
Nursing working experience		
0–1 year	18	16.1
1–2 years	15	13.4
2–3 years	9	8.0
3–5 years	18	16.1
5 years above	52	46.6
ICU license		
Yes	82	73.2
No	30	26.8

Note: Abbreviations and definition: N (Nurse) has clinical work experience of <1 year; N1 (Nurse 1) has clinical work experience of ≥1 year and can perform patients’ basic nursing care; N2 (Nurse 2) has clinical work experience of ≥2 years and participates and implements critically ill patients’ care; N3 (Nurse 3) has clinical work experience of ≥3 years and is able to perform holistic care for critically ill patients/teach and assist nursing quality improvement; N4 (Nurse 4) has clinical work experience of ≥4 years and is able to teach and implement quality administration. Additionally, each level of qualified nurse must pass the certification by Taiwan Nurses Association (TWNA).

**Table 2:** Univariate analysis of the two groups’ demographic variables (*n* = 112).

	Novice Nurses (*n* = 95)	Advanced Nurses (*n* = 17)	t/X^2^	*p*
Variables	n (%)	n (%)		
Age (M ± SD)	27.66 ± 2.56	42.47 ± 3.41		
Age			45.35	0.00
20–29	74 (78)	0 (0)		
30–39	13 (14)	7 (41)		
40–49	7 (7)	6 (35)		
50–59	1 (1)	4 (24)		
Gender			−1.15	0.007
Male	7 (7)	0 (0)		
Female	88 (93)	17 (100)		
Marital status			−9.06	0.00
Single	90 (95)	6 (35)		
Married	5 (5)	11 (65)		
Education Background			6.12	0.04
College	10 (11)	1 (6)		
University	82 (86)	13 (76)		
Master	3 (3)	3 (18)		
Nursing working experience			14.48	0.00
<2 years	33 (35)	0 (0)		
3–5 years	27 (28)	0 (0)		
>5 years	35 (37)	17 (100)		
ICU working experience			23.12	0.00
<3 years	47 (49)	0 (0)		
>3 years	48 (51)	17 (100)		
ICU license			2.77	0.00
Yes	65 (68)	17 (100)		
No	30 (32)	0 (0)		

**Table 3:** Comparison of the differences between novice and advanced nurses at each time point.

		Total (*n* = 112)	Advanced Nurses (*n* = 17)	Novice Nurses (*n* = 95)	*p*
Times	Variables	(M ± SD)	(M ± SD)	(M ± SD)	
T1	PADIS score	40.70 ± 16.66	52.76 ± 10.33	40.33 ± 16.44	0.006
	Knowledge	17.3 ± 8.04	23.11 ± 5.27	17.23 ± 7.95	0.013
	Attitudes	9.48 ± 3.78	11.41 ± 2.73	9.48 ± 3.75	0.11
	Skills	13.88 ± 6.06	18.23 ± 3.57	13.62 ± 5.98	0.002
T2	PADIS Score	63.54 ± 11.66	66.88 ± 8.72	62.77 ± 11.28	0.64
	Knowledge	29.57 ± 5.30	31.47 ± 4.63	29.15 ± 5.05	0.93
	Attitudes	12.94 ± 2.38	13.47 ± 1.73	12.86 ± 2.35	0.53
	Skills	20.97 ± 5.67	21.94 ± 3.05	20.80 ± 5.62	0.55
T3	PADIS Score	65.91 ± 6.34	66.82 ± 6.88	65.75 ± 6.27	0.83
	Knowledge	30.64 ± 3.30	31.00 ± 3.74	30.57 ± 3.24	0.28
	Attitudes	13.44 ± 1.27	13.76 ± 1.39	13.38 ± 1.25	0.92
	Skills	21.83 ± 2.53	22.05 ± 2.53	21.78 ± 2.55	0.50

**Table 4:** Differences between novice and advanced nurses by Repeated measure ANOVA.

	(Novice Nurses–Advanced Nurses) ^a^	SE	t	95% Confidence Interval		*p*-Value
				Lower Bound	Upper Bound	
PADIS total score						
Pre-test	−12.428	4.136	−3.005	−20.624	−4.232	0.003
1st post-test	−4.103	2.883	−1.423	−9.816	1.610	0.157
2nd post-test	−1.066	1.676	−0.636	−4.388	2.257	0.526
Knowledge score						
Pre-test	−5.886	2.008	−2.931	−9.865	−1.907	0.004
1st post-test	−2.313	1.316	−1.757	−4.922	0.296	0.082
2nd post-test	−0.421	0.874	−0.482	−2.153	1.311	0.631
Attitude score						
Pre-test	−1.928	0.954	−2.020	−3.818	−0.037	0.046
1st post-test	−0.607	0.600	−1.012	−1.797	0.582	0.314
2nd post-test	−0.375	0.337	−1.115	−1.042	0.292	0.267
Skills score						
Pre-test	−4.576	1.505	−3.040	−7.559	−1.593	0.003
1st post-test	−1.133	1.405	−0.806	−3.916	1.651	0.422
2nd post-test	−0.261	0.675	−0.387	−1.598	1.076	0.700

^a^ Differences estimated by using repeated-measure ANOVA in a general linear model (GLM) after adjusting for age and marital status.

## Data Availability

Not applicable.

## References

[1] Smithburger P.L., Patel M.K. (2019). Pharmacologic considerations surrounding sedation, delirium, and sleep in critically Ill adults: A narrative review. J. Pharm. Pract..

[2] Pandharipande P.P., Patel M.B., Barr J. (2014). Management of pain, agitation, and delirium in critically ill patients. Pol. Arch. Med. Wewn..

[3] Rowley-Conwy G. (2017). Critical care nurses’ knowledge and practice of delirium assessment. Br. J. Nurs..

[4] Alsulami G., Marie Rice A., Kidd L. (2019). Prospective repeated assessment of self-reported sleep quality and sleep disruptive factors in the intensive care unit: Acceptability of daily assessment of sleep quality. BMJ Open.

[5] Wu C.M., Lai F.-C., Chaou C.-H., Tung H.-H., Maio N.-F. (2016). An exploration of the pain management knowledge of emergency room staffs and factors of influence. J. Nurs..

[6] Eskandari F., Abdullah K.L., Zainal N.Z., Wong L.P. (2017). Use of physical restraint: Nurses’ knowledge, attitude, intention and practice and influencing factors. J. Clin. Nurs..

[7] Krugman M., Smith K., Goode C.J. (2000). A clinical advancement program: Evaluating 10 years of progressive change. J. Nurs. Adm..

[8] Pierson M.A., Liggett C., Moore K.S. (2010). Twenty years of experience with a clinical ladder: A tool for professional growth, evidence-based practice, recruitment, and retention. J. Contin. Educ. Nurs..

[9] Burket T.L., Fermlee M., Greider P.J., Hippensteel D.M., Rohrer E.A., Shay M.L. (2010). Clinical ladder program evolution: Journey from novice to expert to enhancing outcomes. J. Contin. Educ. Nurs..

[10] Moore A., Meucci J., McGrath J. (2019). Attributes of a successful clinical ladder program for nurses: An integrative review. Worldviews Evid.-Based Nurs..

[11] Weng Y.H., Chen C., Kuo K.N., Yang C.-Y., Lo H.-L., Chen K.-H., Chiu Y.-W. (2015). Implementation of evidence-based practice in relation to a clinical nursing ladder system: A national survey in Taiwan. Worldviews Evid.-Based Nurs..

[12] Lin M., Chen F.C., Kuo C.H., Lin P.C., Cheng H.R., Chiu Y.-W. (2004). The exploration of nurses’ knowledge, attitude, behavior and their influencing factors on N3 case report of the clinical ladder program. Tzu Chi. Med. J..

[13] Messmer P.R., Jones S.G., Taylor B.A. (2004). Enhancing knowledge and self-confidence of novice nurses: The“ Shadow-A-Nurse” ICU program. Nurs. Educ. Perspect..

[14] Serafin L., Pawlak N., Strząska-Kliś Z., Bobrowska A., Czarkowska-Pączek B. (2021). Novice nurses’ readiness to practice in an ICU: A qualitative study. Nurs. Crit. Care.

[15] Santana-Padilla Y.G., Santana-Cabrera L., Bernat-Adell M.D., Linares-Pérez T., Alemán-González J., Acosta-Rodríguez R.F. (2019). Training needs detected by nurses in an intensive care unit: A phenomenological study. Enferm. Intensiva Engl. Ed..

[16] Barr J., Frazer G.L., Puntillo K., Ely E.W., Gelinas C., Dasta J.F., Davidson J.E., Devlin J.W., Kress J.P., Joffe A.M. (2013). Clinical practice guidelines for the management of pain, agitation, and delirium in adult patients in the intensive care unit. Crit. Care Med..

[17] Devlin J.W., Skrobik Y., Gelinas C., Needham D., Slooter A.J.C., Pandharipande P.P., Watson P.L., Weinhouse G.L., Nunnally M.E., Rochwerg B. (2018). Clinical practice guidelines for the prevention and management of pain, agitation/sedation, delirium, immobility, and sleep disruption in adult patients in the ICU. Crit. Care Med..

[18] Khoundabi B., Ansari A.F., Hashemian S.M. (2019). Impact of PAD guideline on masih daneshvari hospital ICU. Biom. Biostat. Int. J..

[19] Hermes C., Acevedo-Nuevo M., Berry A., Kjellgren T., Negro A., Massarotto P. (2018). Gaps in pain, agitation and delirium management in intensive care: Outputs from a nurse workshop. Intensive Crit. Care Nurs..

[20] Tsang J.L., Ross K., Miller F., Maximous R., Yung P., Marshall C., Camargo M., Fleming D., Law M. (2019). Qualitative descriptive study to explore nurses’ perceptions and experience on pain, agitation and delirium management in a community intensive care unit. BMJ Open.

[21] Benner P. (1982). From novice to expert. Am. J. Nurs..

[22] Guidelines for the Nursing Ladder System Planning of the Clinical Professional Competence for Primary Nursing Staff. https://www.twna.org.tw/WebPad/WebPad.aspx?1x8jyHnXeNSHfBGHev4mkg%3D%3D.

[23] Min A.-R., Kim I.S. (2013). Relationship of perception of clinical ladder system with professional self-concept and empowerment based on nurses’ clinical career stage. J. Korean Acad. Nurs. Adm..

[24] Ajri-Khameslou M., Najafi M., Karimollahi M. (2021). Vigilance in Nurses Working in Intensive Care Units. Open J. Nurs..

[25] Li Y.-H., Chou M.-C., Lin L.-D., Tsai C.-C., Lin M.-H. (2022). Relationships between willingness to participate in the nursing clinical ladder program and its related factors among clinical nurses. Healthcare.

[26] Wang J., Xiao Q., Zhang C., Jia Y., Shi C. (2020). Intensive care unit nurses’ knowledge, attitudes, and perceived barriers regarding early mobilization of patients. Nurs. Crit. Care.

[27] Tan C.M., Camargo M., Miller F., Ross K., Maximous R., Yung P., Marshall C., Fleming D., Law M., Tsang J.L. (2019). Impact of a nurse engagement intervention on pain, agitation and delirium assessment in a community intensive care unit. BMJ Open Qual..

[28] Eskandari F., Abdullah K.L., Zainal N.Z., Wong L.P. (2018). The effect of educational intervention on nurses’ knowledge, attitude, intention, practice and incidence rate of physical restraint use. Nurse Educ. Pract..

[29] Ramoo V., Adu H., Rai V., Singh S.K.S., Baharudin A.A. (2018). Educational intervention on delirium assessment using confusion assessment method-ICU (CAM-ICU) in a general intensive care unit. J. Clin. Nurs..

[30] Yeh S.H., Hsiao C.Y., Ho T.H., Chiang M.C., Lin L.W., Hsu C.Y., Lin S.Y. (2004). The effects of continuing education in restraint reduction on novice nurses in intensive care units. J. Nurs. Res..

[31] Chyan M., Chen Y.-C., Guo R.-M., Lee Y.-W. (2004). The effects of education intervention on nurses’ knowledge, attitude, and behavior of restrains in the intensive care units. Chang. Gung Nurs..

[32] Burry L., Hutton B., Williamson D.R., Mehta S., Adhikari N.K., Cheng W., Ely E.W., Egerod I., Fergusson D.A., Rose L. (2019). Pharmacological interventions in the treatment of delirium in critical adults. Cochrane Database Syst. Rev..

[33] Lange S., Mędrzycka-Dąbrowska W., Friganovic A., Oomen B., Krupa S. (2022). Non-pharmacological nursing interventions preventing delirium in ICU patients—Umbrella review with implications for evidence-based practice. J. Pers. Med..

[34] Lange S., Mędrzycka-Dąbrowska W., Friganovic A., Oomen B., Krupa S. (2021). Delirium in patients with critical disease and potential role of thiamine therapy in prevention and treatment: Results of scoping review with implications for evidence-based practice. Int. J. Environ. Res. Public Health.

[35] Park S.-Y., Lee H.-B. (2019). Prevention and management of delirium in critically ill adult patients in the intensive care unit: A review based on the 2018 PADIS guidelines. Acute Crit. Care.

[36] Xu C., Chen Z., Zhang L., Guo H. (2022). Systematic review and meta-analysis on the incidence of delirium in intensive care unit inpatients after cognitive exercise intervention. Ann. Palliat. Med..

